# Characterization of *Pasteurella multocida* type B capsule biosynthesis proteins

**DOI:** 10.1128/iai.00049-26

**Published:** 2026-08-06

**Authors:** Thomas R. Smallman, Chantelle M. Cairns, Frank St. Michael, Xiaochu Wang, Manon Sabouraud, Andrew D. Cox, Marina Harper, John D. Boyce

**Affiliations:** 1 Infection Program, Monash Biomedicine Discovery Institute, Department of Microbiology, Monash University, Victoria, Australia; 2 Vaccine and Emerging Infections Research, Human Health Therapeutics Research Centre, National Research Council, Ottawa, Ontario, Canada; Tsinghua University, Beijing, China

**Keywords:** *Pasteurella multocida*, hemorrhagic septicemia, capsule, capsule biosynthesis

## Abstract

*Pasteurella multocida* causes several severe animal diseases, including hemorrhagic septicemia in ungulates. Strains of *P. multocida* are differentiated into five capsular and nine lipopolysaccharide genotypes. To date, only *P. multocida* strains that produce capsule types B and E have been recovered from cases of hemorrhagic septicemia. The structure of the type B capsule has recently been determined to be a repeating main chain of N-acetyl glucosamine (GlcNAc) and N-acetyl mannosaminuronic acid (ManNAcA), with a fructose and glycine side chain. In this study, we investigated the role of proteins involved in capsule biosynthesis in *P. multocida* strain M1404, a bison hemorrhagic septicemia isolate that produces a type B capsule. Using capsule absorbance assays, Alcian blue stains, and capsule Western blots to assess capsule production and export, as well as in-source collision-induced dissociation mass spectrometry to determine capsule structure, we show that BcbC is likely the capsule synthase; BcbE and BcbG are involved in fructose side chain addition, and BcbH is likely the glycine transferase. Furthermore, we show that inactivation of *bcbE*, *bcbG*, or *bcbH* significantly decreases overall capsule production in *P. multocida* strain M1404. Additionally, heterologous expression of *bcbABCDI* in a *P. multocida* strain VP161 (type A) capsule mutant allowed production of a repeating GlcNAc-ManNAcA monomer in this strain background that was indistinguishable from the type B main chain, indicating that these genes are necessary and sufficient for synthesis of the main repeating type B polysaccharide.

## INTRODUCTION

*Pasteurella multocida* is part of the normal bacterial flora in the upper respiratory tract of many mammal and bird species (1). *P. multocida* can also cause several serious diseases, including fowl cholera, porcine atrophic rhinitis, bovine respiratory disease, and hemorrhagic septicemia (HS) in ungulates (1). Strains of *P. multocida* are often classified by their capsule and lipopolysaccharide (LPS) genotypes, with five capsule and nine LPS genotypes currently identified (2–4). Individual strains produce one of five distinct capsular polysaccharides (namely A, B, D, E, and F) and one of at least 16 distinct LPS structures (2–4). Individual diseases caused by *P. multocida* are often associated with just a subset of capsule and LPS types (1). One of the most important diseases caused by *P. multocida* is HS, a systemic and often rapidly fatal disease of ungulates that results in major economic burden for many countries in Asia and Africa. All HS isolates examined to date have a type B or E capsule genotype, and an LPS L2 genotype (5).

Capsule is an important *P. multocida* virulence factor, with acapsular strains unable to cause disease in animals (6, 7). *P. multocida*, like several other gram-negative bacterial species, produces a capsule using an ABC transport-dependent mechanism (8, 9). This involves synthesis of capsule polymers on short 3-deoxy-D-manno-oct-2-ulosonic acid (Kdo) linker molecules that are attached to phosphatidylglycerol in the inner membrane (8, 9). The capsule is then exported to the surface via a cognate ABC transport system (8, 9). Different *P. multocida* capsule types are composed of different glycosaminoglycan polymers (10–12). The *P. multocida* type B and E capsules are structurally related, with both composed of repeating N-acetyl glucosamine (GlcNAc) and N-acetyl mannosaminuronic acid (ManNAcA) monomers. However, the type B capsule has [-4)GlcNAc(β1-3)ManNAcA(β1-]n linkages, and the type E capsule has [−3)GlcNAc(β1-3)ManNAcA(β1-]n linkages (12). Additionally, the type B capsule has fructose attached to the C3 position and glycine attached to the C6 position of the ManNAcA monomers, while type E has a fructose attached to the C4 position of the ManNAcA monomers and does not have any glycine addition (12).

The genes required for *P. multocida* capsule production are all located within a single locus that contains three regions (13, 14). Region 1 genes encode the ABC transport system; region 2 genes encode the proteins involved in sugar monomer production and type-specific polysaccharide synthesis; and region 3 genes encode the proteins involved in Kdo linker synthesis (13, 14). Genes in regions 1 and 3 are conserved across all *P. multocida* strains, irrespective of capsule type, while genes in region 2 differ by type, resulting in the production of different polysaccharides (13, 14). In genotype B and E strains, there are nine genes in region 2 (12, 13), with four of these genes being unique to each type (12). Previous bioinformatic analyses allowed prediction of putative functions for four of the proteins encoded in region 2 of the M1404 (type B) capsule locus. BcbA and BcbB are homologs of WecB and WecC in *Escherichia coli*, respectively (13). WecB and WecC are involved in mannosaminuronic acid production, suggesting BcbA and BcbB are required for production of the UDP-ManNAcA in *P. multocida*. A glycosyltransferase domain was identified in BcbC (13), suggesting that BcbC is the capsule synthase. Comparison of *P. multocida* and *Haemophilus influenzae* strains that produce similar capsule structures, but with different monomer linkages and side chains, suggested that BcbE, BcbF, and BcbG were involved in fructose addition, and BcbH in glycine addition (12). However, an M1404 *bcbH* mutant showed reduced virulence in mice and significantly reduced immunofluorescence when incubated with capsule type B polyclonal antiserum, suggesting that the *bcbH* mutant did not produce capsular polysaccharide (15).

Given the importance of *P. multocida* capsule for host predilection and disease, understanding how the type B and E capsules are synthesized and their role in pathogenesis and host interactions is crucial knowledge for the development of novel treatments. In this study, we investigated the role of different proteins encoded in region 2 of the *P. multocida* strain M1404 capsule locus. We showed that disrupting *bcbC* abolished capsule production, indicating that BcbC is the likely type B capsule synthase. Inactivation of *bcbE* and *bcbG* resulted in a polysaccharide lacking fructose and glycine, and inactivation of *bcbH* resulted in a polysaccharide lacking glycine. Furthermore, the *bcbE*, *bcbG*, and *bcbH* mutants showed significantly reduced overall capsule production. Finally, we showed that heterologous production of BcbABCDI in a *P. multocida* VP161 *hyaD* mutant allowed production of a GlcNAc-ManNAcA capsule lacking side chain additions, confirming that these proteins are necessary and sufficient for synthesis of the main chain polysaccharide.

## RESULTS

### BcbC is essential for capsule production

Initially, we predicted the putative function of BcbC by identifying conserved domains, predicting protein structure, and identifying proteins with structural similarity. Searches against the NCBI conserved domain database identified two domains in BcbC, a glycosylase family A (GT-A)/glycosyltransferase family 2 domain at amino acids 342–448 and a glycosylase type 4/WbuB-like domain at amino acids 681–1,000. Furthermore, the predicted BcbC 3D structure generated by AlphaFold 3 contained two separated domains (Fig. S1A). The predicted BcbC structure between amino acids 314 and 638 aligned closely with the crystal structure of TarS 1-339 from *Staphylococcus aureus* (PDB100 accession number 5TZ8, 0.99 angstroms over 127 matched pairs; Fig. S1B). Additionally, the predicted BcbC structure between amino acids 640 and 1,033 aligned closely with the crystal structure of PimB′ from *Corynebacterium glutamicum* (PDB100 accession number 3OKA, 1.32 angstroms over 73 matched pairs; Fig. S1C). Both TarS and PimB′ have been shown to be glycosyltransferases (16, 17), indicating that BcbC contains two distinct glycosyltransferase domains.

**Fig 1:**
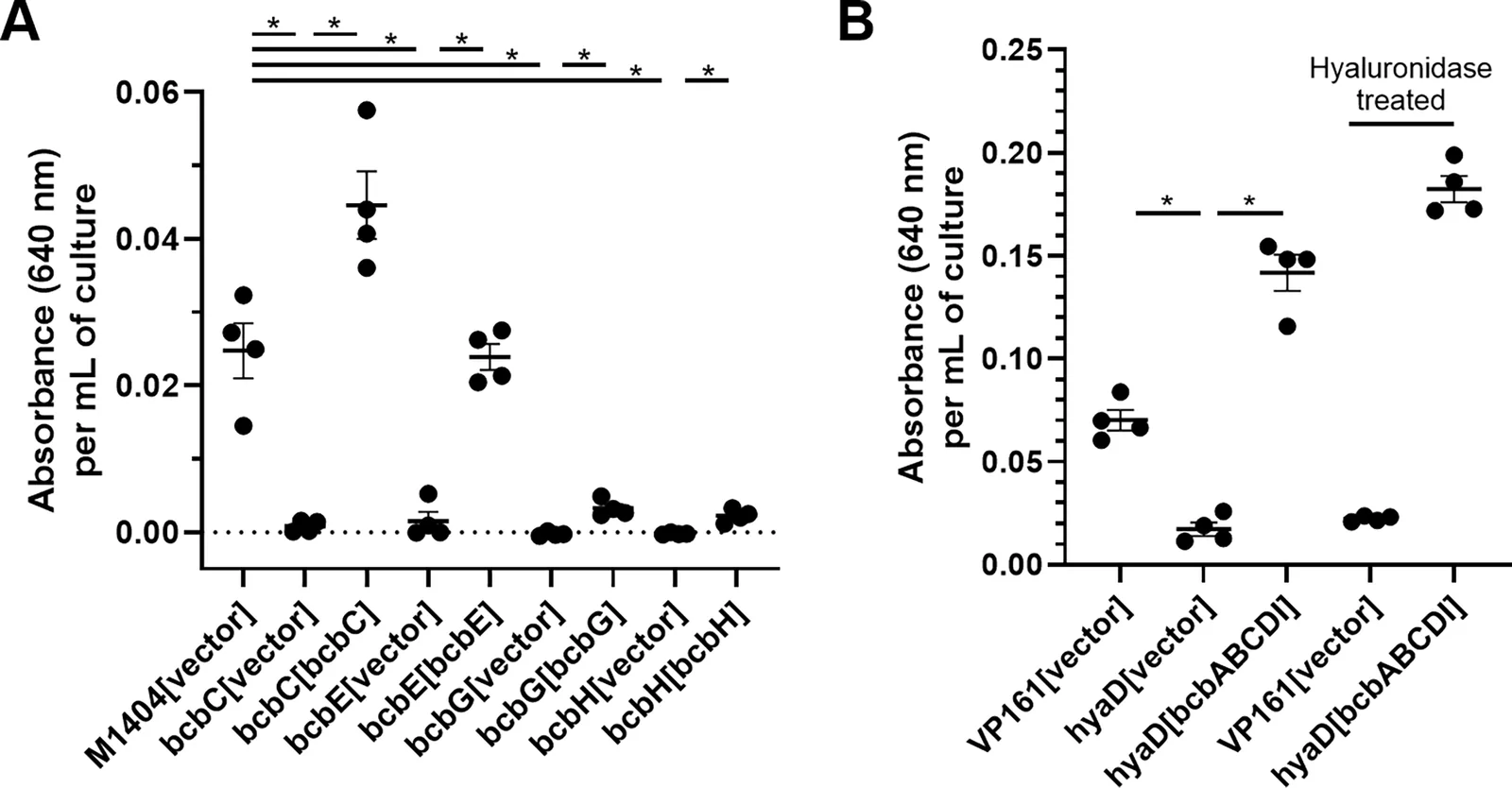
Capsule production by *P. multocida* strains used in this study. Capsule production was measured using direct capsule absorbance assays, where the surface-extracted capsule was mixed with a Stains-All solution and the absorbance was measured at 640 nm. (**A**) Assay performed using capsule extracted from wild-type M1404 harboring pAL99S, the different M1404 capsule mutants harboring pAL99S or the appropriate complementation plasmid following overnight growth on horse blood agar. (**B**) Assay performed using capsule extracted from wild-type VP161 harboring pAL99S only, a VP161 *hyaD* mutant provided with pAL99S, or the *bcbABCDI* expression plasmid following growth to mid-exponential phase in heart infusion broth. Capsule extracted from wild-type VP161 harboring pAL99S and the *hyaD* mutant with the *bcbABCDI* expression plasmid were also treated with hyaluronidase for 30 min prior to measuring absorbance. Differences in capsule production were assessed using Mann-Whitney *U*-tests, with error bars representing mean ± SEM. *P < 0.05.

**Fig 2:**
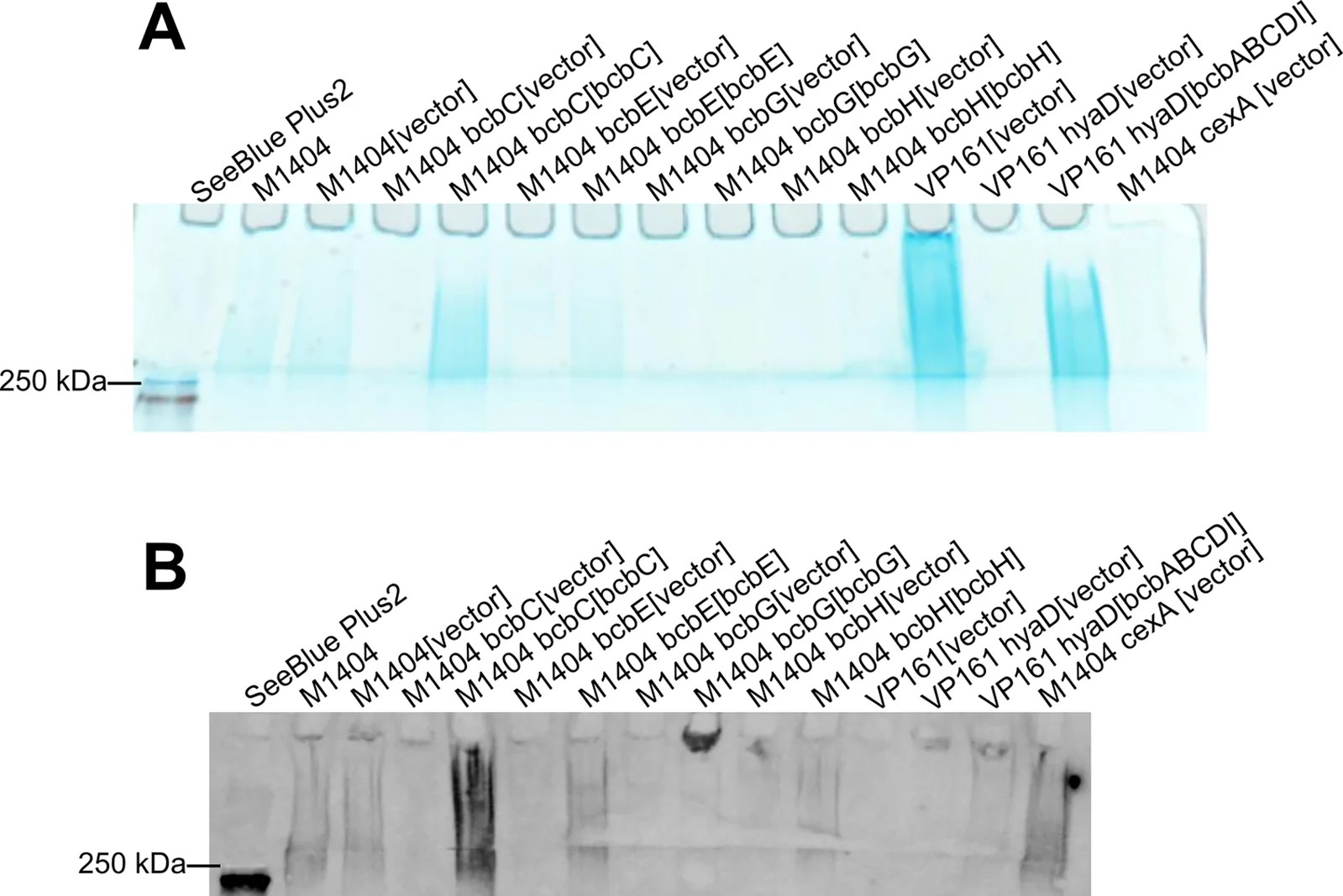
Assessing capsule production and export using Alcian blue staining and capsule-specific Western immunoblotting. Both capsule and whole cell lysate prepared from strains grown overnight on heart infusion agar. Extracellular extracted capsule and whole cell lysates were loaded onto polyacrylamide gels (5% stacker and 12.5% resolving layer) and electrophoresed at 200 V for 50 min. (**A**) Surface-attached capsule was visualized by staining with Alcian blue stain (125 µg/mL). (**B**) Whole cell lysate samples were transferred to a 0.45 µm nitrocellulose membrane, and the capsule was visualized using rabbit anti-type B antisera and a goat antirabbit antibody conjugated to Alexa Fluor 488. For both Alcian blue and capsule-specific Western immunoblotting, the samples were run alongside the SeeBlue Plus2 prestained Protein Standard, with the 250 kDa marker position shown at the left of each panel.

To investigate whether BcbC was the capsule synthase as predicted, we inactivated *bcbC* in *P. multocida* strain M1404 using a modified version of the mobile group II intron system known as ClosTron (18) (Table S1). A single intron insertion in *bcbC* was confirmed by Sanger sequencing using genomic DNA as a template with an outward-firing intron-specific primer (Table S2). To generate the complemented strain, the *bcbC* mutant was provided with a wild-type copy of *bcbC* on the expression vector pAL99S (Table S1). Additionally, the *bcbC* mutant was also transformed with pAL99S empty vector to generate an empty vector control strain (Table S1). Capsules were extracted from the surface of cells representing the above strains and assessed by capsule absorbance assays and Alcian blue stains. The total capsule, including the intracellular capsule that was produced but not exported to the surface, was assessed by Western blots using whole cell lysate and rabbit anti-type B capsule antisera. For both the Alcian blue stain and capsule Western blots, an M1404 *cexA* mutant, which has an active capsule synthesis pathway but an inactive capsule ABC transport system, was included as a control that showed capsule production but no export to the surface. The capsule absorbance assays showed that the *bcbC* mutant produced barely detectable absorbance compared to M1404 harboring pAL99S, indicative of a highly reduced capsule on the *bcbC* mutant cell surface (Fig. 1A). Providing the *bcbC* mutant with a wild-type copy of *bcbC* in *trans* restored absorbance to above wild-type levels (Fig. 1A). The Alcian blue stain of the surface-extracted capsule showed a high-molecular-weight product above the 250 kDa marker for wild-type M1404 and M1404 harboring pAL99S, showing the capsule is present on the surface of these cells (Fig. 2A). In comparison, the M1404 *cexA* mutant harboring pPBA1100, which has previously been shown to lack capsule on the surface of the cell (6), had no capsular material visible in the Alcian blue stain (Fig. 2A). Inactivation of *bcbC* resulted in no high-molecular-weight product being detected in the Alcian blue stain, indicating that no capsule is present on the cell surface of this mutant. Importantly, capsule production was restored to levels above that observed for the wild-type in the *bcbC* mutant provided with a wild-type copy of *bcbC* in *trans* (Fig. 2A). Western blotting, using type B capsule antiserum, showed the presence of immunoreactive material in the whole cell lysates of wild-type M1404, M1404 harboring pAL99S, and the M1404 *cexA* mutant harboring pPBA1100, indicating that these strains could produce type B capsular polysaccharide (Fig. 2B). The whole cell lysate of the *bcbC* mutant showed no immunoreactive material, suggesting no capsular polysaccharide production in this strain. Providing the *bcbC* mutant with a functional copy of *bcbC* in *trans* restored capsule production to levels above that observed in the wild-type strain (Fig. 2B). Collectively, these data show that wild-type M1404, the M1404 wild-type harboring pAL99S, and the complemented *bcbC* mutant were able to produce and export capsule, that inactivation of *cexA* did not abrogate type B capsule production but stopped export and that inactivation of *bcbC* abrogated capsule production in M1404.

**Fig 3:**
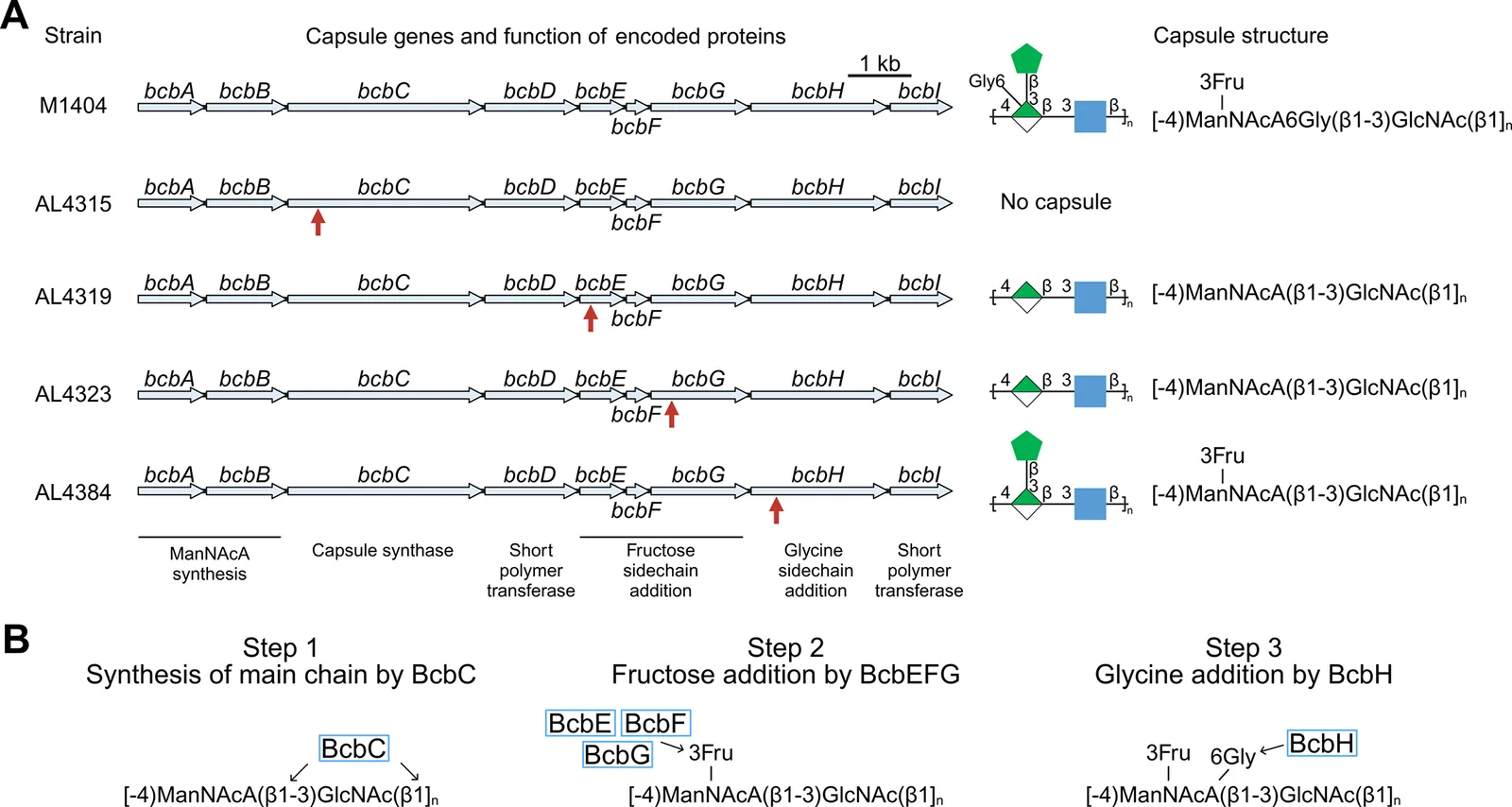
(**A**) The capsule biosynthesis locus and predicted capsule structure produced by wild-type *P. multocida* strain M1404 and various M1404 capsule mutant strains analyzed in this study. The intron insertion site in each mutant strain is shown by a red arrow. The predicted capsule structure produced by each strain is derived from the IS-CID data (see Table 1 and Fig. S2 to S6). (**B**) Predicted steps for capsule synthesis in M1404. Proteins involved in each step are shown as blue boxes.

Electrospray ionization-mass spectrometry (ESI-MS) analysis was performed using the intact capsular material recovered from wild-type M1404, M1404 harboring pAL99S, the *bcbC* mutant harboring pAL99S, and the complemented *bcbC* mutant. To obtain detailed structural information, in-source collision-induced dissociation (IS-CID) was used to fragment the polysaccharide using a high-voltage cone (19). The IS-CID analysis of wild-type M1404, M1404 harboring pAL99S, and the complemented *bcbC* mutant identified the notable ions *m*/*z* 275.5^+^ (HexNAcA and Gly) and 478.3^+^ (HexNAcA, Gly, and HexNAc), consistent with the type B capsule (Table 1; Fig. S2). None of these ions indicative of type B-like capsular material were identified in the IS-CID analysis of samples from the *bcbC* mutant (Table 1; Fig. S3). These data support the Alcian blue and Western blotting data that inactivation of *bcbC* abolishes capsule production and, together with the bioinformatics analyses strongly suggest that BcbC is the capsule synthase (Fig. 3).

**TABLE 1:** Composition of capsule produced by the wild-type *P. multocida* strain M1404 and various mutants and complemented strains^*a*^

Strain	Genotype/sample	Notable ions [M^+^] (Da)	Proposed composition^*b*^
pBA814	M1404	275.5 478.3 662.1^*c*^ 681.2^*c*^	HexNAcA, Gly HexNAcA, Gly, HexNAc HexNAcA, Gly, HexNAc, Hex, Na HexNAcA, Gly, 2HexNAc
AL4221	M1404 (vector)	274.9 421.2 443.2 478.3 605.5^*c*^ 624.6^*c*^ 660.6^*c*^ 662.1^*c*^ 681.2	HexNAcA, Gly HexNAcA, HexNAc HexNAcA, HexNAc, Na HexNAcA, Gly, HexNAc HexNAcA, HexNAc, Hex, Na HexNAcA, 2HexNAc 2HexNAcA, HexNAc, Na HexNAcA, Gly, HexNAc, Hex, Na HexNAcA, Gly, 2 HexNAc
AL4315	M1404 *bcbC*::intron (vector)	None	None
AL4317	M1404 *bcbC*::intron (*bcbC*)	275.6 421.4 478.3 660.2^*c*^ 681.3	HexNAcA, Gly HexNAcA, HexNAc HexNAcA, Gly, HexNAc 2HexNAcA, HexNAc, Na HexNAcA, Gly, 2HexNAc
AL4319	M1404 *bcbE*::intron (vector)	421.2 624.3 646.3 660.2^*c*^	HexNAcA, HexNAc HexNAcA, 2HexNAc 2HexNAcA, HexNAc 2HexNAcA, HexNAc, Na
AL4321	M1404 *bcbE*::intron (*bcbE*)	275.2 421.2^*c*^ 478.2 662.2^*c*^	HexNAcA, Gly HexNAcA, HexNAc HexNAcA, Gly, HexNAc HexNAcA, Gly, HexNAc, Hex, Na
AL4323	M1404 *bcbG*::intron (vector)	421.2 624.2 646.2 660.2^*c*^	HexNAcA, HexNAc HexNAcA, 2HexNAc 2HexNAcA, HexNAc 2HexNAcA, HexNAc, Na
AL4325	M1404 *bcbG*::intron (*bcbG*)	274.9 421.2 478.3 605.7^*c*^	HexNAcA, Gly HexNAcA, HexNAc HexNAcA, Gly, HexNAc HexNAcA, HexNAc, Hex, Na
AL4384	M1404 *bcbH*::intron (vector)	421.4^*c*^ 443.3 605.1	HexNAcA, HexNAc HexNAcA, HexNAc, Na HexNAcA, HexNAc, Hex, Na
AL4385	M1404 *bcbH*::intron (*bcbH*)	275.3 421.2 478.2 605.2^*c*^	HexNAcA, Gly HexNAcA, HexNAc HexNAcA, Gly, HexNAc HexNAcA, HexNAc, Hex, Na
AL4537	VP161 *hyaD*::intron (*bcbABCDI*)	421.2 624.6 660.2^*c*^	HexNAcA, HexNAc HexNAcA, 2HexNAc 2HexNAcA, HexNAc
	Hyaluronic acid	203.9 380.2 402.3^*c*^ 583.7^*c*^ 605.7^*c*^	HexNAc HexNAc, HexA HexNAc, HexA, Na 2HexNAc, HexA 2HexNAc, HexA, Na

^*a*^ Positive ion electrospray-mass spectrometry data of cell capsular material isolated from the strains detailed below. For each strain, the notable ions observed and the proposed composition for those ions are detailed.

^*b*^ Gly, glycine (57.1 Da); Hex, hexose (162.2 Da); HexNAc, N-acetylhexosamine (203.2 Da); HexNAcA, hexosaminuronic acid (217.2 Da); Na, sodium (23.0 Da).

^*c*^ Minor peak.

There was variation in glycine addition observed for strain M1404 harboring pAL99S. Identification of the ions *m*/*z* 275.5^+^ and 478.3^+^ in the IS-CID analysis of capsular material from M1404 harboring pAL99S showed the presence of glycine addition; however, identification of the notable ions *m*/*z* 421.2^+^ (HexNAcA, HexNAc) and 443.2^+^ (HexNAcA, HexNAc, and Na) from capsular material recovered from M1404 harboring pAL99S suggests that glycine was not always present on the ManNAcA in this strain (Fig. S2). As such, in the following analyses, we defined a strain as having no glycine addition only if the IS-CID data had complete loss of the ions characteristic of glycine addition.

### Inactivation of *bcbE*, *bcbG*, or *bcbH* results in modified capsule structure and nearly complete loss of capsule production in *P. multocida* strain M1404

Based on previous comparative bioinformatic analyses, BcbE, BcbF, and BcbG were predicted to be involved in the addition of the fructose moiety, and BcbH was predicted to be involved in the addition of the glycine moiety (12). To investigate the role of these proteins, we generated individual insertional (group II intron) mutants for *bcbE*, *bcbG*, and *bcbH*, as well as corresponding empty vector and complemented strains (Table S1). Mutagenesis of *bcbF* was attempted, but no mutants were able to be generated. Capsule production and the structure of capsules produced by the *bcbE*, *bcbG*, and *bcbH* mutants and complemented strains were assessed as described above. Capsule absorbance assays showed the *bcbE*, *bcbG*, and *bcbH* mutants produced significantly reduced absorbance readings and therefore extracellular capsule compared to wild-type M1404 (Fig. 1A). Providing the *bcbE* mutant with a wild-type copy of *bcbE* in *trans* restored capsule production to wild-type levels (Fig. 1A). Although statistically significant, providing the *bcbG* and *bcbH* mutants with a wild-type copy of the corresponding gene only partially restored extracellular capsule levels (Fig. 1A). The Alcian blue showed that the *bcbE*, *bcbG*, and *bcbH* mutants harboring pAL99S had barely detectable capsules on the cell surface (Fig. 2A). As measured by Alcian blue reactivity, providing the *bcbE* mutant with *bcbE* in *trans* restored cell surface capsule levels. However, providing the *bcbG* and *bcbH* mutants with a wild-type copy of the corresponding gene did not restore cell surface capsule levels (Fig. 2A). The anti-type B capsule Western blots showed that there was almost no intracellular immunoreactive capsule produced by the *bcbE*, *bcbG*, and *bcbH* mutants harboring pAL99S (Fig. 2B). Whole cell lysate samples from the complemented *bcbE* and *bcbH* mutants showed the presence of immunoreactive capsular material, while the complemented *bcbG* mutant had only very high-molecular-weight immunoreactive material in the whole cell lysate (Fig. 2B). Collectively, these data indicate that disrupting *bcbE*, *bcbG*, or *bcbH* significantly reduces total capsule production.

IS-CID analysis of the capsular material recovered from the *bcbE* and *bcbG* mutants gave a spectrum with the *m*/*z* ions 421.2^+^ (HexNAcA and HexNAc), 624.3^+^ (HexNAcA and 2HexNAc), and 646.3^+^ (2HexNAcA and HexNAc) (Table 1 and Fig. S4 and S5), consistent with a structure consisting of a GlcNAc-ManNAcA main chain that lacked both the fructose and glycine side chains. Further evidence supporting side chain loss was the lack of the observed *m*/*z* ions 275.2^+^ (HexNAcA and Gly), 605.5^+^ (HexNAcA, HexNAc, Hex, and Na), and 662.2^+^ (HexNAcA, Gly, HexNAc, Hex, and Na) that were observed in the IS-CID analysis of the capsule produced by wild-type M1404 and M1404 harboring pAL99S. The ions *m*/*z* 275.2^+^, 605.7^+^, and 662.2^+^ were identified in the spectra generated using capsular material recovered from the complemented *bcbE* and *bcbG* mutant strains, showing restoration of glycine and fructose addition to the capsule produced by each of the complemented strains. IS-CID analysis of the capsular material produced by the *bcbH* mutant showed that the capsule lacked the *m*/*z* 275.2^+^ (HexNAc-Gly) and 478.3^+^ (HexNAcA, Gly, and HexNAc) ions (Fig. S6), indicating loss of the glycine side chain. Furthermore, the capsule still produced the ion *m*/*z* 605.1^+^ (HexNAcA, HexNAc, Hex, and Na), indicative of a fructose addition without glycine. Analysis of the capsule produced by the complemented *bcbH* mutant detected ions at *m*/*z* 275.2^+^ and 478.3^+^, indicative of restoration of the glycine addition. These data indicate that BcbH is essential for the addition of glycine to the capsule polymer and that in the absence of glycine, fructose addition can still occur (Fig. 3). Taken together, these data also support the prediction that BcbE and BcbG are required for the addition of the fructose side chain and that without fructose addition, glycine addition does not occur. Furthermore, the low levels of total (surface and intracellular) capsular material produced by each of the *bcbE*, *bcbG*, and *bcbH* mutants suggest that the side chain additions are required for efficient synthesis of the polysaccharide.

### A *P. multocida* strain VP161 *hyaD* mutant expressing *bcbABCDI* produces type B-like main chain capsule

The data above indicate that *bcbE*, *bcbG*, and *bcbH* encode proteins involved specifically in the addition of the fructose and glycine side chains to the main chain polymer. We previously predicted that the other genes in region 2 of the M1404 capsule biosynthesis locus encoded proteins that were required for activated UDP-ManNAcA monomer biosynthesis (*bcbA* and *bcbB*), addition of ManNAcA and GlcNAc monomers to the Kdo linker (*bcbD* and *bcbI*), and polysaccharide polymerization (*bcbC*) (9, 13, 14). To confirm that these genes were sufficient for the production of the type B main chain polymer, we cloned *bcbABCDI* from strain M1404 into the expression plasmid pAL99S. We then used the *bcbABCDI* expression plasmid to transform a *P. multocida* VP161 *hyaD* mutant. *P. multocida* strain VP161 typically produces a hyaluronic acid (type A) capsule; however, inactivation of *hyaD*, which encodes a hyaluronic acid synthase, abolishes capsule production (20). The VP161 *hyaD* mutant has intact copies of all the highly conserved capsule locus region 1 and region 3 genes required for production of the ABC transport system (*hexABCD*) and Kdo linker synthesis (*phyAB*), and as such should allow capsule biosynthesis at the inner membrane and export out of the cell.

Wild-type VP161 harboring pAL99S and the VP161 *hyaD* mutant provided with pAL99S or the plasmid encoding *bcbABCDI* were each assessed for capsule production as described above. The capsule absorbance assays showed that the VP161 *hyaD* mutant had significantly lower absorbance than wild-type VP161, while providing the *hyaD* mutant with *bcbABCDI* in *trans* significantly increased the absorbance compared to the *hyaD* mutant harboring empty pAL99S (Fig. 1B). To determine if the measured capsule represented hyaluronic acid or a type B-specific polysaccharide, capsule extracted from wild-type VP161 or the *hyaD* mutant provided with *bcbABCDI* was pretreated with hyaluronidase, an enzyme that specifically degrades hyaluronic acid, prior to the addition of Stains-All and absorbance reading. Hyaluronidase pretreatment of capsule extracted from VP161 harboring pAL99S significantly reduced the absorbance reading of the extracellular material (Fig. 1B). Comparatively, the hyaluronidase pretreatment had no effect on the absorbance reading generated from the capsule extracted from the *hyaD* mutant harboring the *bcbABCDI* expression plasmid (Fig. 1B), indicating that the heterologous expression of *bcbABCDI* in this strain resulted in the production of a non-hyaluronic acid capsular polysaccharide. Furthermore, the Alcian blue stain of extracted material showed the presence of surface-exposed capsular material recovered from the VP161 harboring the pAL99S strain and the *hyaD* mutant harboring the *bcbABCDI* expression plasmid, but no surface capsular polysaccharide in the *hyaD* mutant harboring pAL99S (Fig. 2A). The capsule-specific Western blot analysis showed that no type B capsule was present in the whole cell lysates of VP161 harboring pAL99S and the *hyaD* mutant harboring pAL99S, but there was a capsule reactive to type B antisera present in the whole cell lysate of the *hyaD* mutant harboring the *bcbABCDI* expression plasmid (Fig. 2B). IS-CID analysis of the capsular material produced by the *hyaD*[*bcbABCDI*] strain identified ions at *m*/*z* of 421.2^+^ (HexNAcA and HexNAc), 624.6^+^ (HexNAcA and 2HexNAc), and 842.1^+^ (2HexNAcA and 2HexNAc), which are indicative of a GlcNAc-ManNAcA polysaccharide and represent the main chain polymer of the type B capsule (Table 1; Fig. S7). The ions identified were identical to those identified from the capsule produced by the M1404 *bcbE* and *bcbG* mutants and different from ions identified from the IS-CID analysis of purified hyaluronic acid (Table 1; Fig. S7). Collectively, these data show that heterologous expression of *bcbABCDI* in a VP161 *hyaD* mutant allowed production of a capsular polysaccharide similar to the main chain type B polysaccharide.

## DISCUSSION

In this study, we assessed the role of different proteins encoded in region 2 of the type B capsule biosynthesis locus in the bovine HS isolate M1404. It is important to note that the capsule structures in this study were determined using IS-CID data, which can generate complicated spectra. Therefore, the IS-CID data were assessed in combination with the type B capsule structure determined previously using nuclear magnetic resonance spectroscopy (12), which allowed for prediction of capsule structure in each strain.

Disruption of *bcbC* in strain M1404 abolished capsule production and export, as shown by capsule absorbance assays, Alcian blue stains, type B capsule-specific Western blotting, and IS-CID analysis. The bioinformatic analysis also showed that the two domains present in BcbC each had structural homology with two different glycosyltransferases, TarS and PimB′ (16, 17). The *P. multocida* capsule type A, D, and F synthases (HyaD, DcbF, and FcbD, respectively) all contain two glycosyltransferase domains and have been shown to add both monomers to the growing capsule polymer (21–23). Given that bioinformatic analysis of BcbC identified two glycosyltransferase domains that had structural homology with known glycosyltransferases and disrupting *bcbC* halted capsule synthesis, we suggest that BcbC is the type B capsule synthase. The data presented here do not conclusively rule out the possibility that BcbC is required for ManNAcA synthesis, as disrupting this activity would also abrogate capsule production. However, BcbA and BcbB are homologs of WecB and WecC, respectively, and WecB and WecC are produced by some Enterobacteriaceae species that produce enterobacterial common antigen (ECA), with ManNAcA being a repeating subunit of ECA (24). WecB is a UDP-N-acetylglucosamine 2-epimerase that converts UDP-GlcNAc to UDP-ManNAc, and WecC is a UDP-N-acetylmannosamine dehydrogenase that converts UDP-ManNAc to UDP-ManNAcA (25, 26). This indicates that BcbA and BcbB are likely required for UDP-ManNAcA synthesis in *P. multocida* capsule type B strains.

BcbE, BcbG, and BcbH were shown to be important for the addition of the fructose and glycine side chains, as had previously been predicted (12), and each of these genes was important for wild-type levels of capsule production. IS-CID analysis indicated that the *bcbE* and *bcbG* mutant strains produced a capsule that lacked both the glycine and fructose side chain additions, while the *bcbH* mutant produced a capsule that only lacked the glycine addition (Fig. 3). These data suggest that BcbH adds the glycine side chain and that both BcbE and BcbG are required for the addition of the fructose side chain. The lack of both glycine and fructose additions to the capsule in both the *bcbE* and *bcbG* mutants suggests that BcbH requires the acceptor molecule to have a fructose side chain, which in turn suggests that the order of capsule synthesis is as follows: the assembly of the GlcNAc-ManNAcA main chain polymer, followed by addition of the fructose, and then lastly, the addition of glycine (Fig. 3B). The Western blots showed that the *bcbE*, *bcbG*, and *bcbH* mutants had almost completely abrogated capsule production. Despite this, enough material was recovered to perform IS-CID analysis. The capsule extraction process used for IS-CID analysis can lyse cells, and it is therefore likely that the IS-CID samples include both intracellular and cell surface capsular material. Capsule polymers identified in the IS-CID samples derived from the *bcbG* and *bcbH* mutants are likely to be intracellularly derived, given the lack of a capsule on the surface of the cell.

Complementation of the *bcbE*, *bcbG*, and *bcbH* mutants with their respective single genes restored the capsule structure to that of wild-type M1404, as measured by IS-CID. However, each complementation had different effects on the amount of capsule produced. Complementation of the *bcbE* mutant restored surface capsule production to wild-type levels, whereas complementation of the *bcbG* and *bcbH* mutants did not significantly increase surface or intracellular capsule production. Investigation of protein localization in *E. coli* has shown that ABC transport-dependent capsule biosynthesis systems likely form a complex at the inner membrane. Membrane fractionation and analysis of proteins from an *E. coli* K5 strain showed that several capsule biosynthesis proteins are associated with the inner membrane (27), and MS analysis of cross-linked proteins from an *E. coli* K1 strain found several capsule biosynthesis proteins cross-linked to each other (28). As such, if a similar membrane complex is formed in *P. multocida* M1404, then altering the level of individual capsule biosynthesis proteins may impact the stoichiometry of an inner membrane complex and result in overall reduced capsule production.

Heterologous production of BcbABCDI from M1404 in a VP161 *hyaD* mutant allowed synthesis of a HexNAc-HexNAcA polymer that was identical to the type B capsule main chain in the IS-CID data and was also insensitive to hyaluronidase treatment. The amount of capsule produced by this strain was similar to that produced by wild-type VP161, suggesting that the absence of side chains does not impede type B capsule main chain production or extracellular transport in this heterologous background. Given that BcbA and BcbB are likely required for ManNAcA synthesis and BcbC is likely the capsule synthase, we predict that BcbD and BcbI are the transferases that add a short capsule polymer onto the Kdo linker. Production of a surface-exposed type B-like capsule in a type A background strain also showed that the ABC transport systems (HexABCD and CexABCD) and the Kdo linker synthases (PhyAB and LipAB) are functionally interchangeable between *P. multocida* type A and B strains that produce different capsule types. We attempted to investigate whether the fructose and glycine additions could also occur if the entire region 2 from M1404 was expressed in the *hyaD* mutant. Initially, all region 2 genes from M1404 were cloned into a low-copy number *E. coli*-specific pWSK29-based expression plasmid. This was followed by the introduction of a *P. multocida* origin of replication (*oriV*) and a kanamycin resistance gene. However, when this plasmid was used to transform the VP161 *hyaD* mutant, no transformants were recovered, despite successful transformation of the empty vector.

Interestingly, separate inactivation of *bcbE*, *bcbG*, or *bcbH* almost completely abrogated capsule production in the native M1404 system, but they were not required for heterologous production of the type B-like main chain polymer in the VP161 *hyaD* mutant. Supporting our analyses, inactivation of *bcbH* in M1404 has been shown previously by immunofluorescence to have an undetectable capsule on the cell surface (15). Furthermore, our recent TraDIS analysis of an M1404 mutant library recovered from systemic infections in mice showed that the entire capsule locus, including each of *bcbE*, *bcbG*, and *bcbH*, was required for survival *in vivo* (29). These data indicate that the entire type B capsule locus is required for M1404 to produce a surface-exposed capsule. Further work to elucidate the differences in capsule production in the native and heterologous expression backgrounds is warranted.

This study represents the first report of a genetic interchange of capsule type between *P. multocida* strains. The strains used in these exchange experiments, M1404 and VP161, produce structurally distinct capsules and cause different diseases in two distinct animal classes. M1404 is a bovine HS strain isolated from bison in North America, and VP161 is a fowl cholera strain isolated from a chicken in Vietnam (30). Our results pave the way for virulence studies using isogenic strains with different capsule types that will help elucidate the role of capsule type in host predilection and disease syndrome.

## MATERIALS AND METHODS

### Bacterial strains and growth conditions

Bacterial strains and plasmids used in this study are described in Table S1. *P. multocida* strains were cultured in heart infusion broth (Oxoid), on solid heart infusion agar (1.5% wt/vol agar), or on horse blood agar (Columbia blood agar base from Oxoid, defibrinated horse blood from Australian Ethical Biologicals). *E. coli* strains were cultured in lysogeny broth. All strains were grown at 37°C for 16–24 h, with liquid cultures shaken at 200 rpm. When antibiotic selection was required, the appropriate antibiotic was added at the following concentrations: kanamycin 50 µg/mL and spectinomycin 50 µg/mL.

### Bioinformatic analysis of proteins of interest

Domains in proteins of interest were identified using the conserved domain database (31). Putative structures of proteins of interest were generated using AlphaFold 3 (32). The AlphaFold-predicted structures were then used to find proteins with similar structures using Foldseek to search against the PDB100 database (33). Structural alignments between BcbC and proteins of interest were performed using ChimeraX (34), with the alignment score given as angstrom distance over matched amino acid pairs.

### DNA extraction, DNA manipulation, Sanger sequencing, and oligonucleotides

Genomic DNA was extracted using the HiYield Genomic DNA Mini Kit (Real Biotech Corporation), and plasmid DNA was extracted using the NucleoSpin Plasmid Kit (Macherey-Nagel) as per the manufacturer’s instructions. Extracted DNA was assessed using agarose gel electrophoresis and Qubit Fluorometry (Life Technologies). Polymerase chain reactions were performed using either *Taq* DNA polymerase (Roche) or Phusion high-fidelity DNA polymerase (New England Biolabs) as per the manufacturer’s instructions. Restriction digestion was performed using restriction enzymes from New England Biolabs, and ligation was performed using T4 DNA ligase (Roche), as per the manufacturer’s instructions. Sanger sequencing was performed using BigDye Terminator v3.1 Ready Reaction Mix (Applied Biosystems) with genomic DNA or plasmid DNA used as the template. Oligonucleotides used in this study were produced by Sigma-Aldrich and are listed in Table S2.

### Transformation, site-directed mutagenesis, and expression plasmid production

Transformation of *P. multocida* was performed by electroporation, as previously described (7). Site-directed insertional mutagenesis of genes of interest was performed using a modified version of the ClosTron system (18), with pAL953 as the base plasmid, as previously described (35). Briefly, intron insertion sites in genes of interest were identified using the ClosTron intron design tool (18). Splicing by overlap extension PCR was used to generate a modified group II intron targeting region (primers listed in Table S2), which was cloned into the appropriate region of the group II intron in pAL953 (plasmids listed in Table S1). Each mutagenesis plasmid was separately used to transform M1404. Mutants were confirmed to have an intron insertion in the gene of interest by performing PCR using primers that flanked the gene of interest and by direct Sanger sequencing using genomic DNA and the oligonucleotide BAP6544, which anneals to the intron and allows for sequencing across the intron-genomic DNA junction.

For complementation and heterologous expression experiments, we generated expression plasmids using the *P. multocida-E. coli* shuttle vector pAL99S. The wild-type copy of the gene(s) of interest was amplified using wild-type M1404 genomic DNA as the template, with forward and reverse primers that contained appropriate restriction sites for cloning into pAL99S (Table S2). The purified PCR product and pAL99S were individually digested with appropriate restriction enzymes, purified, then ligated together. To generate the *bcbABCDI* expression plasmid, *bcbI* was cloned first into pAL99S to generate the plasmid pAL1882, then *bcbABCD* was cloned upstream of *bcbI* to generate pAL1895. The nucleotide sequence of all cloned fragments and the P*tpiA* promoter region of the expression plasmids was confirmed using Sanger sequencing. Complemented strains were generated by transforming each mutant strain with the appropriate expression plasmid via electroporation. Each mutant was also separately transformed with pAL99S to generate appropriate vector-only control strains (Table S1).

### Capsule absorbance assays

For capsule assays performed using wild-type VP161 and VP161 derivative strains, cells were grown in heart infusion broth to mid-exponential phase growth. For capsule assays performed using wild-type M1404 and M1404 derivative strains, cells were grown overnight on horse blood agar and recovered directly from the plate in 1× phosphate-buffered saline (PBS). Cells were standardized to the same optical density to ensure even input of cells. Capsule absorbance assays were performed as previously described (4). Briefly, *P. multocida* cells were washed, resuspended in 500 µL of PBS and 100 µL of capsule extraction buffer (500 mM citric acid and 1% wt/vol Zwittergent 3-10), and heated at 50°C for 20 min. The capsule was mixed with 5× volume of absolute ethanol, incubated at 4°C for 30 min, and centrifuged at 13,000 × *g* for 20 min, before the supernatant was removed and the capsule was resuspended in dH_2_O. The capsule was mixed with 9× volume of Stains-All solution (200 μg/mL, 50% vol/vol formamide, and 0.06% vol/vol glacial acetic acid), and optical density was measured at 640 nm. For hyaluronidase treatment, the extracted capsule was incubated with 500 units of hyaluronidase (Sigma) for 30 min prior to performing capsule absorbance assays. Significant differences in capsule production between strains were determined using a Mann-Whitney *U*-test.

### Alcian blue staining

The capsule for Alcian blue staining was extracted as described above in the capsule absorbance assay section. Alcian blue staining was performed as previously described (36), with minor modifications. The capsule was loaded onto a 12.5% polyacrylamide gel with a 5% polyacrylamide top layer for resolving the samples and run at 200 V for 50 min. The samples were not boiled prior to loading, and each of the washes with the fixing buffer (25% wt/vol EtOH and 10% wt/vol glacial acetic acid in deionized water) was performed for 15 min at room temperature. The Alcian blue stain used was 0.125% wt/vol in fixing buffer.

### Type B capsule Western blots

Whole cell lysate samples were prepared from cells grown overnight on heart infusion agar. Cells were recovered directly from the plate in 1× PBS, washed twice in 1× PBS, and resuspended to ~4 × 10^8^ CFU in 100 µL. The cells were centrifuged for 5 min at 13,000 × *g* and resuspended in 40 µL of ddH_2_O with 200 ng of Proteinase K (Merck) and incubated at 60°C for 1 h. Cells were mixed 1:1 with sample buffer before being incubated at 99°C for 10 min. The boiled cells were then centrifuged for 10 min at 13,000 × *g* to pellet any insoluble material. The samples were separated using SDS-PAGE with a polyacrylamide gel that had a 5% stacking layer and a 12.5% resolving layer and run at 200 V for 50 min. Electrophoresis samples were transferred to a 0.45 µm nitrocellulose membrane via electroblotting. Type B capsule was detected using polyclonal rabbit antiserum raised against type B capsule (kindly donated by Thula Wijewardana, Veterinary Research Institute, Peradeniya, Sri Lanka) (6), diluted 1 in 10 in 1× PBS, and then visualized following incubation with goat antirabbit IgG conjugated to Alexa Fluor 488 (Thermo Fisher). Immunofluorescence was detected using an Amersham Imager 680 (GE Healthcare).

### Mass spectrometry of capsular polysaccharides

Capsules were extracted from freeze-dried cell pellets representing each strain using 90% phenol as previously described (37). Briefly, the sample was centrifuged at 100,000 × *g* for 5 h, and the resulting supernatant was freeze-dried. The freeze-dried sample was then dissolved in 10 mL of water, precipitated with 3× volumes of methanol, and subsequently precipitated with 0.75× volumes of acetone, as previously described (12). Following dialysis, the freeze-dried sample, containing milligram quantities of capsular material, was then analyzed by MS.

ESI-MS was performed using a Waters SQ Detector 2 instrument. Samples were injected using a mobile phase of 50% acetonitrile in water with 0.1% trifluoroacetic acid, at a flow rate of 50 µL/min. A volume of 10–15 µL of each sample was injected at 1 mg/mL. A 4-min run-time per sample allowed for detection of a sample peak over approximately 15 s, followed by extensive system flushing. In-source fragmentations of the intact polysaccharide were acquired over an *m*/*z* range of 100–1,000 in positive ion mode using a high cone voltage of 160 V to generate fragmentation ions of the polysaccharide.

## Data Availability

Data generated in this study are available from the corresponding author upon request.

## SUPPLEMENTAL MATERIAL

Supplemental material iai.00049-26-s0001.docx Tables S1 and S2; Fig. S1 to S7.

ASM does not own the copyrights to Supplemental Material that may be linked to, or accessed through, an article. The authors have granted ASM a non-exclusive, world-wide license to publish the Supplemental Material files. Please contact the corresponding author directly for reuse.
