# Supplementary material for: Characterization of Pasteurella multocida type B capsule biosynthesis proteins
Source: Infect Immun. 2026 Aug 6;94(9):e00049-26. doi: 10.1128/iai.00049-26 (PMC13552107; doi:10.1128/iai.00049-26)
Supplement: Supplementary file 1 [file iai.00049-26-s0001.docx]

## Supplemental material

Supplemental material I for Characterisation of novel proteins necessary for *Pasteurella multocida* type B capsule biosynthesis

Supplemental tables

**Table S1.** Strains and plasmids used in this study.

| Strain or plasmid | Description | Source or reference |
| --- | --- | --- |
| Strains |  |  |
| *P. multocida* |  |  |
| M1404 | Bison haemorrhagic septicaemia isolate, capsule type B and LPS type L2 | K. R. Rhoades, National Animal Disease Centre, Ames, Iowa |
| VP161 | Fowl cholera isolate, capsule type A and LPS type L1 | (1) |
| AL2188 | VP161 harbouring pAL99S; Spec^R^ | (2) |
| AL3574 | VP161 *hyaD* insertional mutant (intron insertion between nucleotides 1,483-1484) | (3) |
| AL3961 | AL3574 harbouring pAL99S; Spec^R^ | (3) |
| AL4221 | M1404 harbouring pAL99S; Spec^R^ | This study |
| AL4315 | M1404 *bcbC* insertional mutant (intron insertion between nucleotides 1,311-1,312) harbouring pAL99S; Kan^R^ Spec^R^ | This study |
| AL4317 | M1404 *bcbC* insertional mutant (intron insertion between nucleotides 1,311-1,312) harbouring pAL1836; Kan^R^ Spec^R^ | This study |
| AL4319 | M1404 *bcbE* insertional mutant (intron insertion between nucleotides 321-322) harbouring pAL99S; Kan^R^ Spec^R^ | This study |
| AL4321 | M1404 *bcbE* insertional mutant (intron insertion between nucleotides 321-322) harbouring pAL1837; Kan^R^ Spec^R^ | This study |
| AL4323 | M1404 *bcbG* insertional mutant (intron insertion between nucleotides 958-959) harbouring pAL99S; Kan^R^ Spec^R^ | This study |
| AL4325 | M1404 *bcbG* insertional mutant (intron insertion between nucleotides 958-959) harbouring pAL1838; Kan^R^ Spec^R^ | This study |
| AL4384 | M1404 *bcbH* insertional mutant (intron insertion between nucleotides 615-616) harbouring pAL99S; Kan^R^ Spec^R^ | This study |
| AL4385 | M1404 *bcbH* insertional mutant (intron insertion between nucleotides 615-616) harbouring pAL1850; Kan^R^ Spec^R^ | This study |
| AL4537 | AL3574 harbouring pAL1895; Spec^R^ | This study |
|  |  |  |
| *E. coli* |  |  |
| DH5α | F^-^ *deoR endA*1 *gyrA96 hsdR17* (r_K_^-^ m_K_^-^) *recA1 relA1* *supE44* thi-1 Δ(*lacZYAargFV169*) ᶲ80*lacZ*ΔM15 | Bethesda Research Laboratories |
| AL1296 | DH5α harbouring pAL99S; Spec^R^ | (2) |
| AL1995 | DH5α harbouring pAL953; Kan^R^ Spec^R^ | (2) |
|  |  |  |
| Plasmids |  |  |
| pAL99S | *P. multocida­*-*E. coli* expression plasmid; Spec^R^ P*tpiA* | (2) |
| pAL953 | *P. multocida* vector containing group II intron; Spec^R^ Kan^R^ | (2) |
| pAL1804 | pAL953 with group II intron targeted to *bcbC*; Spec^R^ Kan^R^ | This study |
| pAL1824 | pAL953 with group II intron targeted to *bcbE*; Spec^R^ Kan^R^ | This study |
| pAL1826 | pAL953 with group II intron targeted to *bcbG*; Spec^R^ Kan^R^ | This study |
| pAL1836 | *bcbC* amplified from M1404 cloned into pAL99S; Spec^R^ | This study |
| pAL1837 | *bcbE* amplified from M1404 cloned into pAL99S; Spec^R^ | This study |
| pAL1838 | *bcbG* amplified from M1404 cloned into pAL99S; Spec^R^ | This study |
| pAL1849 | pAL953 with group II intron targeted to *bcbH*; Spec^R^ Kan^R^ | This study |
| pAL1850 | *bcbH* amplified from M1404 cloned into pAL99S; Spec^R^ | This study |
| pAL1882 | *bcbI* amplified from M1404 cloned into pAL99S; Spec^R^ | This study |
| pAL1895 | *bcbABCD* amplified from M1404 cloned into pAL1882; Spec^R^ | This study |

**Table S2.** Oligonucleotides used in this study.

| Oligonucleotide | Sequence (5’ – 3’) | Description |
| --- | --- | --- |
| ClosTron group II intron mutagenesis | | |
| BAP6544 | CGAAATTAGAAACTTGCGTTCAGTAAAC | EBS Universal oligonucleotide used to retarget the group II intron and to perform Sanger sequencing to confirm intron insertions |
| BAP9478 | AAAAAAGCTTATAATTATCCTTAGATTTCGTACCAGTGCGCCCAGATAGGGTG | IBS oligonucleotide used to retarget the group II intron in pAL953 to *bcbC* |
| BAP9479 | CAGATTGTACAAATGTGGTGATAACAGATAAGTCGTACCAGATAACTTACCTTTCTTTGT | EBS1d oligonucleotide used to retarget the group II intron in pAL953 to *bcbC* |
| BAP9480 | TGAACGCAAGTTTCTAATTTCGATTAAATCTCGATAGAGGAAAGTGTCT | EBS2 oligonucleotide used to retarget the group II intron in pAL953 to *bcbC* |
| BAP9527 | AAAAAAGCTTATAATTATCCTTACTTTACATTTTTGTGCGCCCAGATAGGGTG | IBS oligonucleotide used to retarget the group II intron in pAL953 to *bcbE* |
| BAP9528 | CAGATTGTACAAATGTGGTGATAACAGATAAGTCATTTTTAATAACTTACCTTTCTTTGT | EBS1d oligonucleotide used to retarget the group II intron in pAL953 to *bcbE* |
| BAP9529 | TGAACGCAAGTTTCTAATTTCGGTTTAAAGTCGATAGAGGAAAGTGTCT | EBS2 oligonucleotide used to retarget the group II intron in pAL953 to *bcbE* |
| BAP9537 | AAAAAAGCTTATAATTATCCTTACAACTCAATCTTGTGCGCCCAGATAGGGTG | IBS oligonucleotide used to retarget the group II intron in pAL953 to *bcbG* |
| BAP9538 | CAGATTGTACAAATGTGGTGATAACAGATAAGTCAATCTTGATAACTTACCTTTCTTTGT | EBS1d oligonucleotide used to retarget the group II intron in pAL953 to *bcbG* |
| BAP9539 | TGAACGCAAGTTTCTAATTTCGGTTAGTTGTCGATAGAGGAAAGTGTCT | EBS2 oligonucleotide used to retarget the group II intron in pAL953 to *bcbG* |
| BAP9612 | AAAAAAGCTTATAATTATCCTTAGGTAACATGATTGTGCGCCCAGATAGGGTG | IBS oligonucleotide used to retarget the group II intron in pAL953 to *bcbH* |
| BAP9613 | CAGATTGTACAAATGTGGTGATAACAGATAAGTCATGATTACTAACTTACCTTTCTTTGT | EBS1d oligonucleotide used to retarget the group II intron in pAL953 to *bcbH* |
| BAP9614 | TGAACGCAAGTTTCTAATTTCGATTTTACCTCGATAGAGGAAAGTGTCT | EBS2 oligonucleotide used to retarget the group II intron in pAL953 to *bcbH* |
|  |  |  |
| Expression plasmid construction | | |
| BAP9481 | ATCATTCCCGGGAGGAGGCAAAACTATGAAAAAGTCAAA | Forward oligonucleotide used for amplification of *bcbC*, contains an XmaI recognition site and consensus ribosome binding site |
| BAP9482 | GAGAAAGTCGACTTATGCATTATTTACGCCTT | Reverse oligonucleotide used for amplification of *bcbC*, contains a SalI recognition site |
| BAP9530 | GAATGTCCCGGGAGGAGGTTGATATGTTTGTTATTCCAA | Forward oligonucleotide used for amplification of *bcbE*, contains a XmaI recognition site and a consensus ribosome binding site |
| BAP9531 | TTTATGGTCGACCTATTTTAATAATGACTGGTA | Reverse oligonucleotide used for amplification of *bcbE*, contains a SalI recognition site |
| BAP9540 | CTAAAACCCGGGAGGAGTAATGGATGATTATCA | Forward oligonucleotide used for amplification of *bcbG*, contains an XmaI recognition site |
| BAP9541 | TAAATCGTCGACCTAAGCCTCTTTCCACATTTT | Reverse oligonucleotide used for amplification of *bcbG*, contains a SalI recognition site |
| BAP9587 | CTTCCTTCAGATAATACGTT | Oligonucleotide used for sequencing of pAL1804 to confirm wild-type *bcbC* sequence |
| BAP9588 | AATGGTGAGCAAGATAATAC | Oligonucleotide used for sequencing of pAL1804 to confirm wild-type *bcbC* sequence |
| BAP9589 | TATTGATGAGTTGGGGTGTA | Oligonucleotide used for sequencing of pAL1804 to confirm wild-type *bcbC* sequence |
| BAP9591 | TTCCTTTATTCGTGCCTTAG | Oligonucleotide used for sequencing of pAL1826 to confirm wild-type *bcbG* sequence |
| BAP9615 | AAAAAACCCGGGGTGGAAAGAGGCTTAGAATGCA | Forward oligonucleotide used for amplification of *bcbH*, contains a XmaI recognition site |
| BAP9616 | AAAAAAGTCGACGAAGATTAGTTTAAAGTTAGGCTTTC | Reverse oligonucleotide used for amplification of *bcbH*, contains a SalI recognition site |
| BAP9642 | TTATACTGATTTATTGCAAAAA | Oligonucleotide used for sequencing of pAL1849 to confirm wild-type *bcbH* sequence |

Supplemental figures

**Fig. S1.** Structure of BcbC from *Pasteurella multocida* strain M1404 as predicted by AlphaFold **(A).** Legend shows predicted local distance difference test score for the BcbC structural prediction. Structural comparison of BcbC^314-638^ (purple) and TarS^1-339^ (orange, PDB100 accession 5TZ8) from *Staphylococcus aureus* that matched with a root mean square deviation of 0.99 angstroms over 127 matched pairs **(B).** Structural comparison of BcbC^640-1,033^ (purple) and PimB’ (green, PDB100 accession 3OKA) from *Corynebacterium glutamicum* that matched with a root mean square deviation of 1.32 angstroms over 73 matched pairs **(C).** Each structure is shown twice, with the structure rotated 180º on the y-axis to show the opposite side.

**Fig. S2.** IS-CID of capsular material extracted from M1404 harbouring pAL99S (AL4221) **(A)** and wild-type M1404 **(B)**.

**Fig. S3.** IS-CID of capsular material extracted from the M1404 *bcbC* mutant harbouring pAL99S (AL4315) **(A)** and the complemented *bcbC* mutant (AL4317) **(B)**.

**Fig. S4.** IS-CID of capsular material extracted from the M1404 *bcbE* mutant harbouring pAL99S (AL4319) **(A)** and the complemented *bcbE* mutant (AL4321) **(B)**.

**Fig. S5.** IS-CID of capsular material extracted from the M1404 *bcbG* mutant harbouring pAL99S (AL4323) **(A)** and the complemented *bcbG* mutant (AL4325) **(B)**.

**Fig. S6.** IS-CID of capsular material extracted from the M1404 *bcbH* mutant harbouring pAL99S (AL4384) **(A)** and the complemented *bcbH* mutant (AL4385) **(B)**.

**Fig. S7.** IS-CID of capsular material extracted from the VP161 *hyaD* mutant harbouring the *bcbABCDI* expression plasmid (AL4537) **(A)** and purified hyaluronic acid (Sigma) **(B)**.

## References

1.	Wilkie IW, Grimes SE, O'Boyle D, Frost AJ. 2000. The virulence and protective efficacy for chickens of Pasteurella multocida administered by different routes. Vet Microbiol 72:57-68.

2.	Harper M, St Michael F, John M, Vinogradov E, Steen JA, van Dorsten L, Steen JA, Turni C, Blackall PJ, Adler B, Cox AD, Boyce JD. 2013. Pasteurella multocida Heddleston serovar 3 and 4 strains share a common lipopolysaccharide biosynthesis locus but display both inter- and intrastrain lipopolysaccharide heterogeneity. J Bacteriol 195:4854-64.

3.	Smallman TR, Williams GC, Harper M, Boyce JD. 2022. Genome-wide investigation of Pasteurella multocida identifies the stringent response as a negative regulator of hyaluronic acid capsule production. Microbiol Spectr 10:e0019522.
